# Exploring the Perceived Usefulness and Ease of Use of a Personalized Web-Based Resource (Care Companion) to Support Informal Caring: Qualitative Descriptive Study

**DOI:** 10.2196/13875

**Published:** 2019-08-20

**Authors:** Amadea Turk, Emma Fairclough, Gillian Grason Smith, Benjamin Lond, Veronica Nanton, Jeremy Dale

**Affiliations:** 1 Unit of Academic Primary Care Coventry United Kingdom; 2 Carers4Carers Warwickshire United Kingdom

**Keywords:** caregivers, information technology, internet

## Abstract

**Background:**

Informal carers play an increasingly vital role in supporting the older population and the sustainability of health care systems. Care Companion is a theory-based and coproduced Web-based intervention to help support informal carers’ resilience. It aims to provide personalized access to information and resources that are responsive to individuals’ caring needs and responsibilities and thereby reduce the burdens associated with caregiving roles. Following the development of a prototype, it was necessary to undertake user acceptability testing to assess its suitability for wider implementation.

**Objective:**

This study aimed to undertake user acceptance testing to investigate the perceived usefulness and ease of use of Care Companion. The key objectives were to (1) explore how potential and actual users perceived its usefulness, (2) explore the barriers and facilitators to its uptake and use and (3) gather suggestions to inform plans for an area-wide implementation.

**Methods:**

We conducted user acceptance testing underpinned by principles of rapid appraisal using a qualitative descriptive approach. Focus groups, observations, and semistructured interviews were used in two phases of data collection. Participants were adult carers who were recruited through local support groups. Within the first phase, think-aloud interviews and observations were undertaken while the carers familiarized themselves with and navigated through the platform. In the second phase, focus group discussions were undertaken. Interested participants were then invited to trial Care Companion for up to 4 weeks and were followed up through semistructured telephone interviews exploring their experiences of using the platform. Thematic analysis was applied to the data, and a coding framework was developed iteratively with each phase of the study, informing subsequent phases of data collection and analysis.

**Results:**

Overall, Care Companion was perceived to be a useful tool to support caregiving activities. The key themes were related to its appearance and ease of use, the profile setup and log-in process, concerns related to the safety and confidentiality of personal information, potential barriers to use and uptake and suggestions for overcoming them, and suggestions for improving Care Companion. More specifically, these related to the need for personalized resources aimed specifically at the carers (instead of care recipients), the benefits of incorporating a Web-based journal, the importance of providing transparency about security and data usage, minimizing barriers to initial registration, offering demonstrations to support uptake by people with low technological literacy, and the need to develop a culturally sensitive approach.

**Conclusions:**

The findings identified ways of improving the ease of use and usefulness of Care Companion and demonstrated the importance of undertaking detailed user acceptance testing when developing an intervention for a diverse population, such as informal carers of older people. These findings have informed the further refinement of Care Companion and the strategy for its full implementation.

## Introduction

### Background: The Burden of Caring and the Potential of Digital Interventions

Informal carers, who provide unpaid physical, practical, and emotional care, play a particularly vital role in supporting the growing older population, of whom an increasing proportion live with multimorbidity, frailty, and other complex health and social care needs [1]. In the United Kingdom, there are an estimated 7.6 million informal carers aged >16 years, with a significant number of these aged >65 years [2]. Collectively, they form an essential part of the social care system that is estimated to save the state £132 billion every year; without it the provision of care would be unacceptably limited or unaffordable [2]. Hence, supporting the sustainability and effectiveness of informal caregiving is of major importance to individuals, families, and the wider society.

The potential of digital technologies to facilitate access to services and information to support health and well-being is becoming ever more recognized [3,4], and digital technologies are increasingly being used by carers to support their caregiving activity and responsibilities [5-7]. Indeed, digital interventions may significantly enhance the carer’s ability to quickly access information and support. However, identifying reliable, current, and easily accessible resources may be time consuming and challenging, especially for those with limited information technology (IT) literacy [8]. Although an online portal that brings together guidance from carer support organizations, information about activities, and social groups is likely to be of considerable use to carers [9], to date an easy-to-navigate program for carers that provides personalized information, resources, and support to address individual needs has been lacking.

### Care Companion—a Coproduced Theory-Based Digital Resource for Unpaid Carers

**Figure 1:**
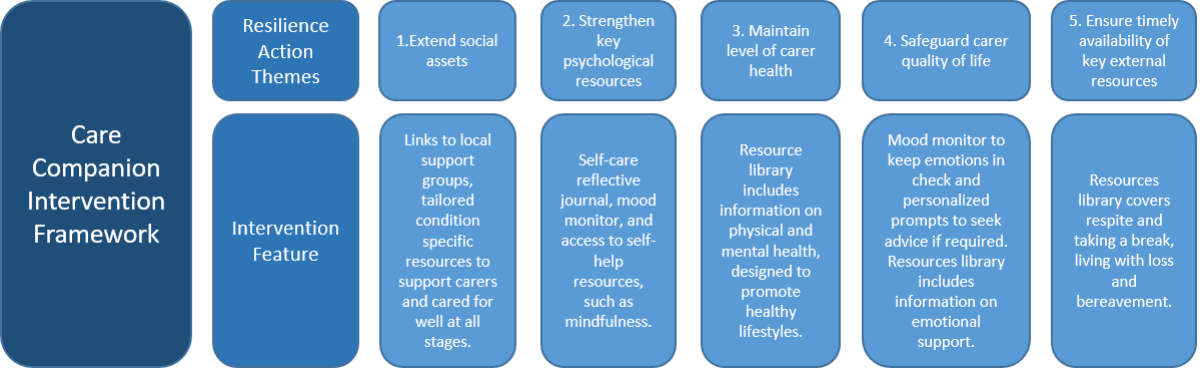
The Care Companion intervention framework.

To help address the need for individually tailored resources, we developed a Web-based platform (Care Companion) to provide profile-driven support to informal carers [10]. The platform was coproduced with older carers and utilized a theory-based approach to support resilience and sustainability and is underpinned by a biopsychosocial model of carer resilience proposed by Parkinson et al [11]. The model comprises 5 independent domains (extending social assets, strengthening psychological resources, ensuring timely availability of key external resources, maintaining physical health, and safeguarding quality of life) that can be targeted to strengthen carer resilience and coping (see Figure 1 for intervention framework) **.** It is recognized that digital interventions that target multiple domains and incorporate a personalized approach that is adaptive to ever-changing needs and issues are more likely to improve carers’ health outcomes [12].

**Figure 2:**
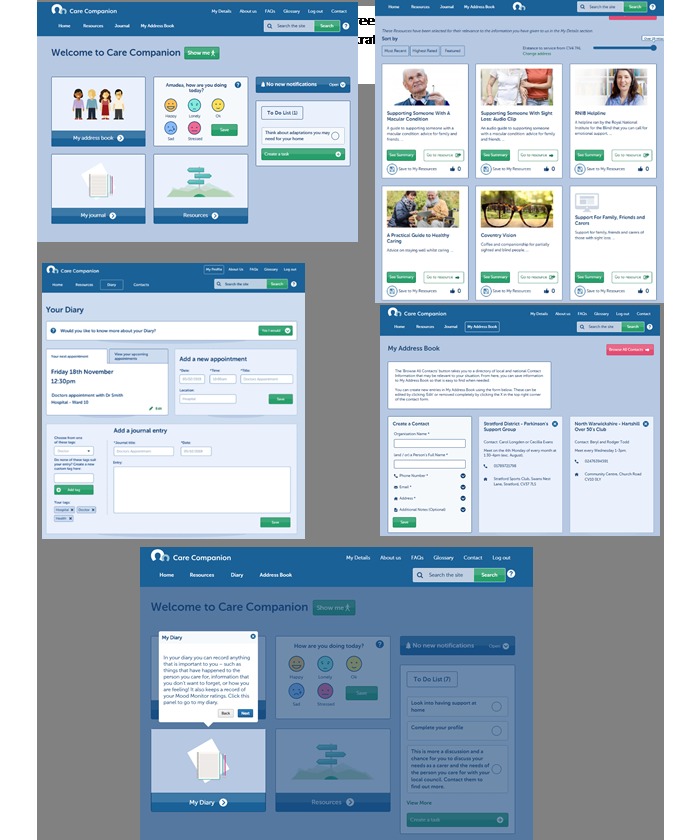
Features of Care Companion: home screen (top) and demonstration of the guided walkthrough available on the site (bottom).

**Figure 3:**
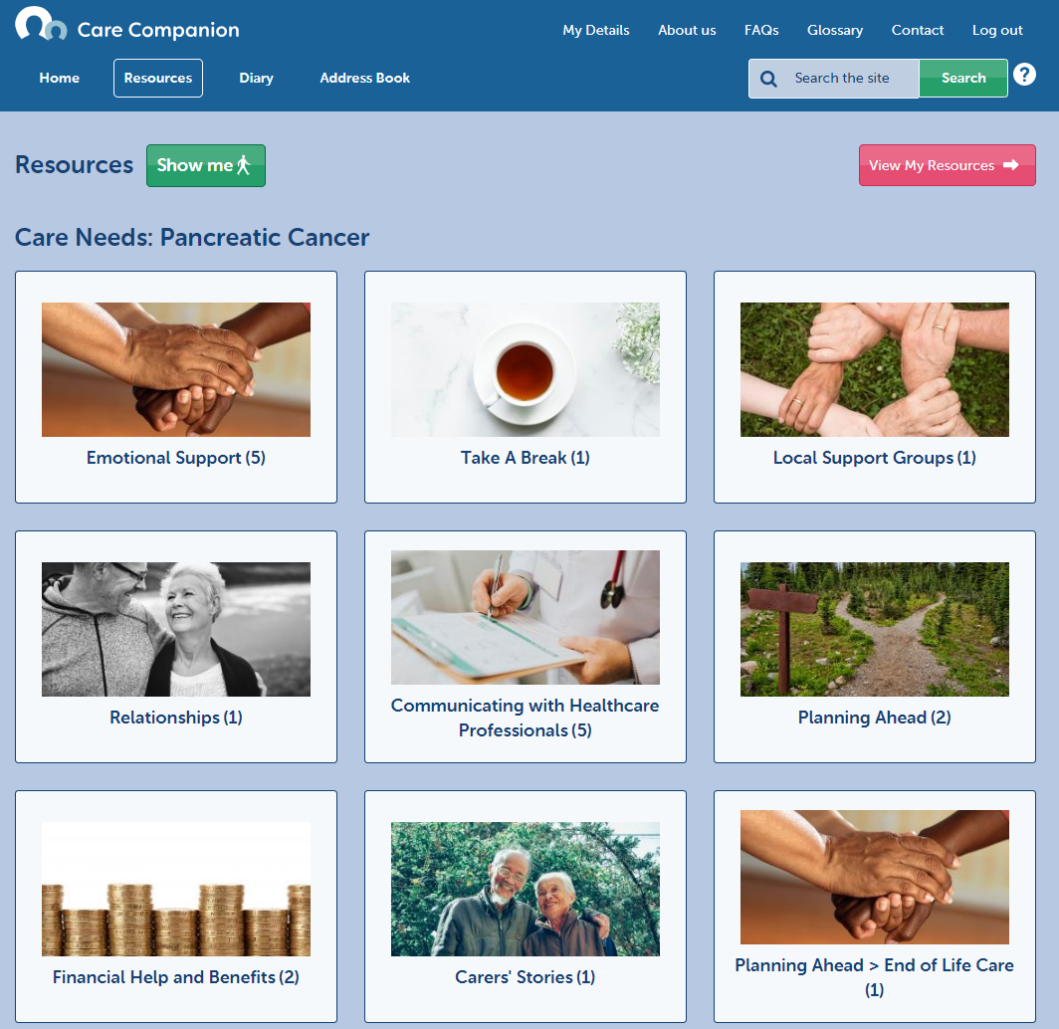
Features of Care Companion: resources.

**Figure 4:**
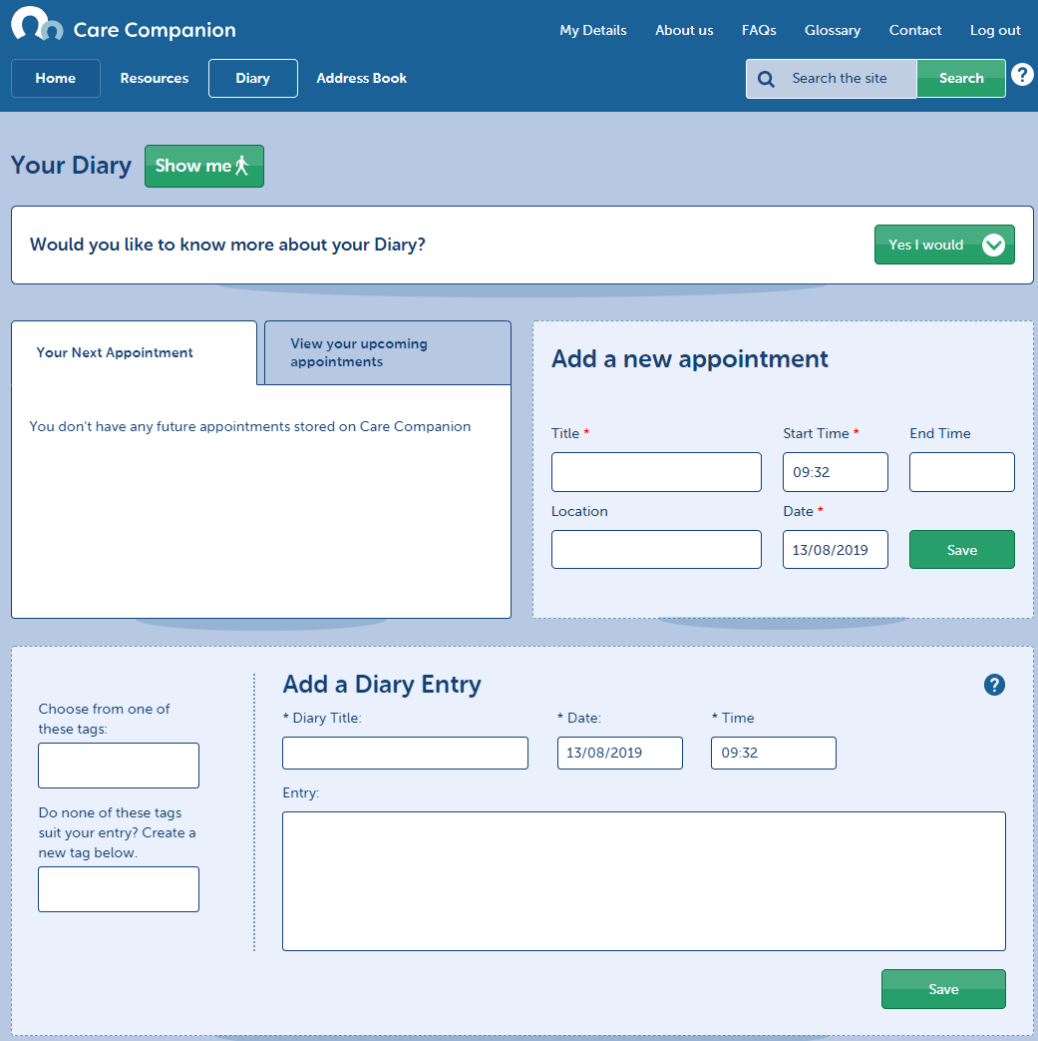
Features of Care Companion: journal.

**Figure 5:**
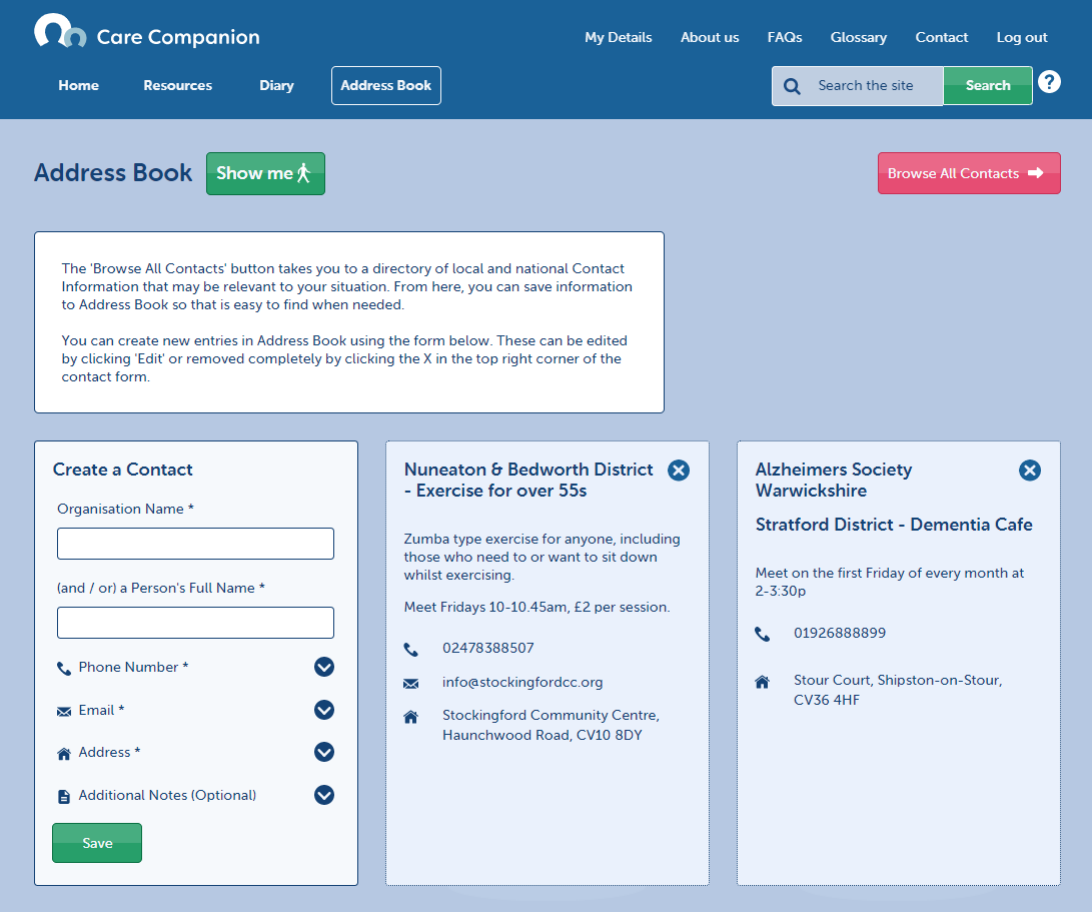
Features of Care Companion: address book, populated with pre-existing contacts of local support groups and with functionality to add own entries.

Care Companion was developed with older carers in mind and includes a guided walk-through of the site, which can be accessed at any time (see Figure 2). It offers links to (1) condition-specific and generic information, local support groups, and other third-sector organizations (see Figure 3), (2) a personal journal for carers to record information, feelings, and thoughts that they deem important (see Figure 4), (3) an address book where carers can save important contacts (see Figure 5), and (4) other features to support self-monitoring (eg, mood of both the carer and the person in their care) [10] (see Figure 2). The resource library targets 3 key areas: carer needs, general information and advice, and sustaining the carer. Users can also access carers’ stories that are designed to promote self-efficacy beliefs through vicarious learning [13].

### Importance of User Acceptability Testing

The number of older people accessing the internet and taking advantage of Web-based services is increasingly rapidly [9,10,13,14]. However, older individuals often have lower levels of confidence in using new technologies compared with younger people [9]. Applying user-centered approaches to understanding the context in which digital health technologies will be used is particularly important when developing interventions for older people [15]. As such, the coproduction of Care Companion was shaped by interviews, focus groups, and workshops with carers [10], underpinned by a theory-driven process of coproduction [16]. This informed its design, content, and implementation.

Having developed a full working version of Care Companion, there was now a need to test user acceptance with a more diverse range of users and stakeholders than those that had participated in the coproduction. User acceptance testing was undertaken to ensure Care Companion’s compatibility with different needs [15] and explore potential barriers and facilitators to its use [17].

Barriers to using Web-based technologies include issues surrounding accessibility, such as the availability of digital devices or internet connections, lack of digital skills, and lack of motivation or awareness of the potential benefits of engaging with technologies. In addition, a lack of trust in digital technologies, such as fear of crime and Web-based scams, concerns relating to privacy, and uncertainty about the credibility of sources of information, may also affect their uptake and use [18,19]. Such barriers may be particularly pertinent to older carers.

Other possible barriers to adoption of Care Companion may include language, culture, and ethnicity. There are estimated to be around 600,000 ethnic minority carers in England and Wales [20], and yet the scope of the initial coproduction had largely excluded consideration of the specific needs associated with culture and ethnicity. The omission of different sociocultural perspectives might inadvertently contribute to inequalities in access to health and social care [21,22]. Hence, user acceptance testing provided an opportunity to explore how different cultural values and norms may influence the uptake and usage of Care Companion.

### Aims and Objectives

The aim of this study was to undertake user acceptance testing to explore the perceived usefulness (how useful the features of the platform are in everyday life) and ease of use of Care Companion and to identify refinements that might be needed before it becomes widely available.

The key objectives were to (1) explore the perceived usefulness and ease of use of Care Companion among the actual and potential users, (2) determine the barriers and facilitators that may affect its uptake and use, including the possible effects of culture and ethnicity, and (3) gather suggestions to inform plans for its wider implementation.

## Methods

### Theoretical Approach

The study was underpinned by the principles of rapid appraisal [23]. This is a pragmatic approach to obtaining information about a specific set of questions within a time and resource-limited real-world setting and has been successfully applied to health services research [24]. It enables rapid assessment of community perspectives of needs and supports translating these findings into action [23].

We adopted a qualitative descriptive approach [25,26] to the analysis of data. Although qualitative description is the least theoretical of qualitative approaches to research [27], it is relevant for generating information about the experience of a specific phenomenon in situations where time and resources are limited [26]. It is less interpretive than other forms of qualitative enquiry as it neither requires the researcher to move far beyond the data nor requires a conceptual or highly abstract rendering of the data [27].

The Unified Theory of Acceptance and Use of Technology (UTAUT) [28] was used to help identify outcomes of interest that are relevant to the adoption and uptake of Care Companion, particularly its technical and practical aspects, as their refinement was recognized as being essential for the platform’s wider launch.

### Study Design

Qualitative methods including focus groups, observations, and semistructured interviews were used to explore key issues surrounding the use and implementation of Care Companion. The study aimed to recruit older carers through purposive sampling. The study was conducted iteratively, with two phases of data collection, each designed to explore different elements of the platform’s use. We aimed to recruit a diverse mix of carers, including individuals from South Asian ethnic backgrounds, from community groups within the local area.

In the first phase, semistructured interviews were undertaken while the carers tried using Care Companion to elucidate how they approached and navigated the site. The second phase involved focus group discussions and participants trialing Care Companion for up to 4 weeks, followed by a semistructured interview in which they shared their experiences of its use. The findings from both phases were used to shape subsequent technical development of Care Companion.

#### Phase 1: Testing Accessibility

Participants were recruited through local carer groups who met regularly and agreed for a member of the research team to drop in during their meetings. Carers were provided with a tablet device and guidance on how to create their profile in Care Companion. They were then interviewed as they navigated the site to (1) understand their interaction with the platform, (2) identify elements that they struggled with, and (3) gain an overall view of their interest and enthusiasm in using it. A *think-aloud* method [30] was used wherein participants were encouraged to voice their thought processes as they navigated the platform to explain why they chose each section. The questions presented to carers while using the site focused on the ease of use, whether the layout was intuitive, whether the appearance was appealing, and ideas on how it could be improved.

Following a brief presentation, participants were asked general questions about their use of digital technology, how they search for information, their initial impressions of Care Companion, which elements they thought would be most useful, and the potential barriers to use. They were then invited to use Care Companion for up to 4 weeks, and those interested were invited to participate in a follow-up telephone interview to discuss their experiences. Further details about how participants from focus groups participated in interviews can be found in Multimedia Appendix 1. The semistructured telephone interviews were planned to last 20 to 30 min and followed a topic guide (see Multimedia Appendix 2 for interview questions) informed by the concepts of the UTAUT [28]. All interviewees signed consent forms.

Table 1 illustrates how the study meets the criteria for rigor defined by Lincoln and Guba [29] using a framework provided by Bradshaw et al [26].

**Table 1:** Demonstrating rigor in exploring the usefulness and ease of use of Care Companion.

Criteria	Definition of criterion	Examples of how criterion is addressed
Credibility	The confidence in the truth of the research findings. Credible and plausible research findings must be drawn from the participants’ original data and need to be correct interpretations of these data.	Trusting relationships between participants and the research team may increase participants’ willingness to share their experiences. A number of steps were taken to build trust: Relationships between the research team and the support groups were developed through past exchange of emails and telephone conversations; The team made use of the Medical School, National Health Service, local authority, and Age UK logos on all communications about the study and on the platform; Furthermore, leading (and trusted) members of the support groups helped arrange focus groups and thus facilitated recruitment; During the focus groups and interviews, the researcher discussed the importance of supporting informal carers, expressing compassion and empathy for those in caring roles Findings from the interviews and focus groups were shared and discussed with the study’s panel of carers that helped verify the findings. Member checking occurred with 1 participant who was interested in being involved in the project long-term. All findings from the study were shared and discussed with its carer advisory panel Data were collected through a variety of methods from people with a range of caring experience. Data analysis was discussed among the members of the research team. These steps aided triangulation Participants were invited to remain engaged by continuing their use of the Care Companion and provided with contact details should they wish to share more feedback with the research team. Some were invited to being involved through membership of the study’s carer panel
Confirmability	The extent to which findings can be confirmed to be real. The extent to which it can be shown that the interpretation of the findings is clearly derived from the data.	Interviews were audio recorded and transcribed verbatim. Transcripts were stored securely on protected computers Notes were taken during focus group discussions and think-aloud interviews An audit trail capturing participant interest, data collection, and the research path was kept Data analysis was conducted in NVivo Direct quotations are used to illustrate the findings and to show that the findings represent the gathered data and are not biased by researchers
Dependability	Establishes whether the study's findings are consistent and repeatable	An audit trail was established describing the study's procedures and progress, including changes that needed to be made during the study
Transferability	The extent to which the findings can be applied to other contexts	The study used purposeful sampling Notes were kept by the researchers during data collection. Researchers were reflexive about their potential impact on the data collection process and other contextual factors

### Ethical Approval

The study received ethical approval from the University of Warwick Biomedical Sciences Research Ethics Committee.

### Analysis

All interviews and focus groups were transcribed verbatim, anonymized, and managed and analyzed using NVivo [31]. Thematic analysis [32] was applied to the data, and a coding framework was developed iteratively during analysis by AT, EF, and BL. This followed a 5-step process of familiarization, identifying a thematic framework, indexing, charting, and mapping and interpretation [28]. Field notes were used to support contextualizing and interpretation of the transcripts—particularly in relation to the think-aloud interviews where participants used and pointed to different aspects of the platform. Coding was conducted inductively [33]. Once coding was complete, key themes were identified, explored, and interpreted by all authors. The analysis of each phase of the study also informed the subsequent phases of data collection and analysis. The final analysis involved synthesizing information from each phase of data collection and integrating the different themes into a broader thematic structure [34].

### Phase 2: Group Discussions, User Acceptability Testing, and Semistructured Interviews

Participants were recruited from local carer groups organized by charities including Parkinson’s UK and a local South Asian carers’ support group. Care group facilitators were contacted through email and asked whether a researcher could join one of their weekly meetings to conduct a focus group.

## Results

### Participant Recruitment

Participants who took part in think-aloud interviews were recruited through local carer support groups. A total of 4 carers with differing levels of IT literacy agreed to participate. They predominantly cared for individuals with neurodegenerative diseases, such as dementia. In total, 4 focus groups were conducted involving a total of approximately 50 participants. Groups varied in size, reflecting the availability and willingness of the different carer groups’ members to participate. Of the participants, 16 expressed an interest in testing Care Companion. Of these, 2 participants declined taking part in a follow-up interview (one told us that they struggled to use the site, and the other felt that the platform did not add to the support they were already receiving). In addition, two other individuals recruited through carer groups volunteered to use Care Companion and participate in a follow-up interview. A total of 10 participants did not respond to the contact made by the research team for follow-up interviews, leaving a total of 6 interviews that were conducted with participants (see Table 2 for further details).

**Table 2:** Summary of participants in user acceptability testing.

Identifier			Recruitment strategy	Gender	Participants, n	Details about caring responsibilities
**Phase 1**						
	**Data collection by think-aloud interview**					
		P1-1	Rural café for supporting older people and their carers	Female	1	Lived separately from person needing care
		P1-2	Rural café for supporting older people and their carers	Female	1	Lived with person needing care
		P1-3	Rural café for supporting older people and their carers	Male	1	Lived with person needing care
		P1-4	Rural café for supporting older people and their carers	Female	1	Lived with person needing care
**Phase 2**						
	**Data collection by focus group**					
		FG 1	Rural café for supporting older people and their carers	3 females	3	All 3 participants were carers for somebody they lived with (parent or spouse).
		FG 2	Local Parkinson disease charity	Large group — mixture of males and females	37	The group was a mixture of people in care and their carers
		FG 3	Local Parkinson disease charity	3 males and 4 females	7	The group was predominantly made up of carers, 1 participant identified as caring for himself
		FG 4	South Asian carer network	1 male and 2 females	3	All lived with person needing care
	**Data collection by interview**					
		P2-1	Referred by carer panel member	Male	1	Living with and caring for spouse for 2 years at the time of the interview
		P2-2	Rural café for supporting older people and their carers	Female	1	Caring for 4 years, providing daily care at the time of the interview
		P2-3	Recruited through invitation sent to dementia support group	Male	1	Living with and caring for spouse for 4 months at the time of the interview
		P2-4	Rural café for supporting older people and their carers	Female	1	Living with and caring for parent for 7 months at the time of the interview
		P2-5	Local Parkinson disease charity	Female	1	Living with and caring for spouse for 6 years at the time of the interview
		P2-6	Local Parkinson disease charity	Male	1	Living with a condition, care for self and support their carer to care for them

### Interview Findings

The key themes identified in the interviews related to the perceived usefulness and ease of use of Care Companion; its appearance and ease of use; the profile setup and log-in process; the safety and confidentiality of personal information; barriers to use and uptake and suggestions for overcoming them; and suggestions for improving Care Companion. Quotes that most clearly illustrated these themes were selected. These are discussed below.

#### Usefulness and Ease of Use of Care Companion

The breadth of available, trustworthy, and bespoke resources and contacts listed on Care Companion (see Figure 2) was rated highly by carers. Participants thought this would make the platform an extremely useful resource for aiding their caregiving. They contrasted this with their experience of using regular search engines that can generate an overwhelming number of results that may not necessarily be relevant. Participants were positive about the fact that this was a resource aimed at carers rather than care recipients. This highlights the scarcity of such resources and that the carer is often overlooked when they are supporting someone with more immediate needs.

I find that by going onto this Care Companion site, there’s a lot of information that can be easy sort of broken down. And you sort of can get to calm down a little bit and think; probably life isn’t quite as bad as you first thought it was, you know. There is help out there. And it triggers it in the right sort of way...it’s got the potential of something being very good. Like I said, with Google, it tends to be a bit overwhelming. With this particular site, it’s tending to hone in and cut down that overwhelmingness.

P2-3

It’s just the way I was thinking about things. I suppose it’s the way my brain is programmed at the moment that everything is for the cared for rather than myself. So, I was thinking that I really should look at it from a totally different angle and use it for my own benefit rather than

P2-2

In contrast, one carer did not participate in a follow-up interview as he felt that Care Companion did not add anything to the support he was already receiving.

The journal feature was received with particular enthusiasm for its potential to log events, appointments, medications, symptoms, and other important aspects of their caring role. It was suggested that this feature would encourage the ongoing use of Care Companion:

I think it would be something that would be very useful, and certainly for me particularly with regards to the journal because at the moment I don’t keep a log of everything that happens. And I do realize now, through just sort of playing around with the package, I do realize just how important that would be to me, to be able to just keep a record

P2-2

I felt that it was a very useful site; I wished I’d known about it 12 months ago. The journal I think will be useful because you could transfer that information to the GP.

P2-1

I think it’s the journal I would probably find most useful, being able to express my feelings, for want of a better expression really.

P2-4

Although Care Companion is aimed at carers, some participants felt that it could also be very useful for the care recipient—to either help themselves or help support their care provider. This was seen as a way of facilitating mutual support to maintain higher levels of independence. It further highlighted the often-blurred boundary between caregiving and self-caring roles, particularly in the early stages of a condition, and that Care Companion should be inclusive in enabling this.

#### Appearance and Ease of Use

Participants were satisfied with the appearance of the site and found it intuitive to use. The headings and signposting within the site were considered to be clear, making its different features easy to find and access (see Figure 2). The guided walk-through was considered to be a useful feature. Some, however, noted that they struggled using it until they had familiarized themselves with the site. Some cited their relative lack of experience with technology as a barrier to easy use:

The appearance was good. The ease of access was alright when I’d learnt how to use it, you know. I’m not a computer expert, but once I’d found my way around yes it’s relatively easy.

P2-1

#### Profile Setup and Log-In Process

A number of key subthemes emerged relating to profile setup and logging in. These include the difficulties of remembering passwords and email addresses and the sensitivity and relevance of profile questions.

##### Remembering Passwords and Email Addresses

Although participants found accessing the platform straightforward, some expressed concern with remembering their log-in details. Indeed, we observed some carers struggling to verify their email when first registering for an account on the platform, either because they were unsure about how to access their email or because they could not remember a password.

###### Two-Factor Authentication

Participants were cautious of Care Companion’s 2-step verification log-in system, where users would input their email and password before getting an automated phone call that delivered a onetime 4-digit code that was needed to allow access. Although 2-step verification was used to help prevent unauthorized log-in to users’ accounts and safeguard their data, participants’ initial views were that this measure was cumbersome. It was, however, accepted that it plays an important role in protecting their information:

I did not like that you needed an automated telephone call to provide a validation code each time you logged in. If someone doesn’t have access to a telephone then they would not be able to use.

FG4, female carer

##### Sensitivity and Relevance of Profile Questions

Some participants noted that certain questions in the profile setup needed further consideration. For instance, at the time of study, the profile questions required carers to comment on their financial situation; it was suggested that this question could be considered stressful for some and that there should be an option to say *unwilling to answer* or *do not know*. Other participants felt that some of the questions were subjective and, therefore, difficult to answer. For example, when rating the independence of the person in their care, one carer noted that this could be difficult to answer. Participants did, however, recognize that the personalization of the resources depended on these questions being answered. Participants also suggested using additional questions to help enhance personalization:

If someone is under pressure, you know, if they've got financial problems then that's just sort of dramatic overload on the issue isn't it, so yes that's relevant.

P1-1

So you might...you might need another saying I don’t know, can’t say or don’t know, or something like that. Do you know what I mean? I mean you could even have a situation where you had a carer who was looking after the person, and their finances were being dealt with by another family member somewhere at the other end of the country.

P1-3

#### Safety and Confidentiality

Participants were concerned about their safety and confidentiality when using Care Companion. They were aware of Web-based scams and expressed concern as to how their information was kept secure, as well as the risks associated with uploading and downloading personal information. Participants were also keen to understand how Care Companion would comply with new general data protection regulation legislation [35] and wanted to know how their information was stored and who would have access to it:

And people should be, it should be explained to people that if they’re going to download it, are you downloading it to a secure place, you know. And give people plenty of prompts to make sure that they could say yes, I’m happy doing this.

P1-3

#### Barriers to Use and Uptake and Suggestions for Overcoming Them

Participants identified a number of issues that could present barriers to the use of Care Companion and its wider implementation. There were concerns expressed about those from lower-socioeconomic backgrounds without access to digital devices being excluded from adopting Care Companion. Other participants noted that some older carers have low levels of digital literacy, which could prevent access to the platform. In addition, South Asian participants highlighted that Care Companion was only available in English, which would hinder access to caregivers with a limited ability to read English. It was also noted that carers of a South Asian background may be hesitant to adopt Care Companion for the fear of how this may impact other statutory support that they are receiving. To this end, they advised that the platform’s purpose as an information and signposting tool be emphasized:

One thing you’ve got to be careful of, for people on benefits or...social housing all that side of things—welfare; that this [Care Companion] has got nothing to do with that. This community of people just in general will be very cagey it if they thought that this was going to impact. So you need to be quite clear in the message that this [Care Companion] is for information purposes only...it wouldn’t affect their care or their rights—it’s just signposting.

FG4, female carer

Participants commented, both in interviews and group discussions, that many carers use devices other than computers to access online services, such as tablets and smartphones. It was suggested that having Care Companion optimized for the use on tablets or smartphones could help overcome some of the extrinsic hardware barriers to accessing the service. In-person training sessions were also recommended to help demonstrate Care Companion and improve the uptake of the platform.

Carers of an Indian background noted that including more visual graphics, such as video to help explain and demonstrate the platform, would bypass the need for verbose text and be helpful for people with limited ability to read English. They also stressed that, for Indian communities, it might be beneficial to adopt a community-driven approach to help spread awareness of Care Companion. This would help enhance carer trust in the platform and thus increase the likelihood of individuals taking-up the service. To this end, promotion of the platform may be advanced by working alongside prominent and well-respected persons and religious groups based in these communities:

I think the only way that you would get other [Indian] people to use it [Care Companion] is perhaps through word-of-mouth...it’s essentially about referring them to this resource...I think that would be essentially the best way to target it to other people.

FG4, female carer

I think it’s about trust. Because if they know you and you say, “oh I used it-it helped me, take a look” I would say well they’ve recommended it, they’ve got reasons behind it...it’s like an added bonus.

FG4, female care

#### Suggestions for Improving Care Companion

Care Companion was still under development at the time of data collection. As a consequence, some participants encountered technical difficulties that have since been resolved. In addition, the carers suggested several improvements to the features of the platform that are summarized in Table 3.

**Table 3:** Summary of suggestions for improvement of Care Companion.

Feature	Suggested improvement
Journal	Add tagging options or subsections that allow users to categorize their entries, and thus enable easier retrieval of information; Improve the ability to search for entries by displaying a calendar; Add the ability to enter events that will occur in the future
Mood monitor	Increase the number of “moods” available, in particular a “stressed” option
Address book	Enhance the personalization of relevant contacts. (In its test format, the contacts list was not as profile-driven as the resources)
Resources	Inclusion of additional links in the resources section to websites that they knew about and thought might be valuable to others
Profile questions	Inclusion of additional profile questions to drive further personalization, such as age categories
Search functionality	In earlier stages of development, the platform’s searching function was limited and often had errors

## Discussion

### Principal Findings

This study has (1) explored the perceived usefulness and ease of use of Care Companion among older carers, (2) identified several barriers and facilitators that may affect its uptake and use, and (3) gathered suggestions for its further refinement and wider implementation. Recruiting different groups of carers to those who had participated in its coproduction [10] helped validate key themes that had been previously identified and also provided new insights. Overall, the carers who participated in our study perceived Care Companion to be a valuable and useful tool to support them in their caregiving activities.

The breadth of personalized, easily accessible, and carer-centered information on a single platform that is easy to navigate was especially celebrated by the participants. In addition to the resources section that had information relevant to supporting and informing caring roles, the journal feature within Care Companion was received with particular enthusiasm. Participants valued its ability to log events and thoughts and other important aspects of their caring role, such as medications, symptoms, and appointments. Facilitators to the uptake and the use of Care Companion were felt to include its simple and intuitive design and the breadth of personalized information. The main barriers to use included low digital literacy, access to digital technologies, the complexity of 2-factor authentication, and an inability to read English.

During the interviews and focus groups, a number of suggestions were voiced to help refine Care Companion. These included enhancing existing features (eg, the addition of tags in journal entries to enable easier retrieval of information) and ways of encouraging wider uptake and use. These included the following suggestions: running brief local training courses to support those with low technological literacy, optimizing the platform for use on devices other than computers (namely tablets and smartphones), and including more visual graphics to mitigate verbose text and the associated language barriers. Finally, suggestions emerged for a more culturally informed strategy to promote Care Companion within Indian communities by way of a community-driven approach to maximize trust in the service.

### Impact of User Acceptability Testing

Both phases of user acceptance testing were used to drive changes to the Care Companion prototype. These changes include the following: enhancements to the journal feature to enable scheduling of appointments and tagging of entries, additional profile questions relating to ethnicity, religion, and culture to drive further personalization of resources, simplification of the 2-step authentication process, and removal of technical difficulties experienced by users.

### Limitations

Carers face a number of burdens, including a lack of free time for themselves [1]. Inevitably, they are a difficult population to recruit for the purposes of research. Therefore, we adopted a highly flexible and opportunistic recruitment strategy that used a range of different interview and observation methods and settings to collect data. The focus groups were based on pre-existing groupings and thus differed widely in size. The strength of this approach was the convenience to participants. However, using pre-existing groups meant that there was little possibility for collecting sociodemographic data on participants, and this inevitably raised questions about representativeness. In addition, in a large focus group, it is not possible to ensure that everyone’s views can be fully heard, and some participants may not have felt confident to express their opinions in front of such a large group.

The study had limited funding and had to be completed within a relatively short timescale to inform the planned area-wide implementation of Care Companion. Hence, it had a relatively small sample size that limits the generalizability and transferability of the findings.

The study aimed to explore how factors, such as culture and ethnicity, may influence the uptake of Care Companion. As this was a rapid and small study, we only targeted local South Asian groups, as these represent the largest ethnic minority groups in England [21,36]. Although we attempted to explore the potential of using Care Companion with South Asian carers, we experienced significant difficulties in recruiting participants. As a result, we were only able to recruit carers of Indian heritage, therefore exploring only their experiences rather than a diversity of South Asian perspectives. This may have been because the research was undertaken during summer months which coincided with holidays and religious events. Furthermore, the researcher (BL) was a male, which may have made it difficult to recruit South Asian female carers [36].

Furthermore, caregiving is understood as an intrinsic part of family duty among many South Asian communities [21,37], meaning that members of these communities may not identify as carers because they locate their caregiving within broader religious and cultural norms [37]. South Asian caregivers may, therefore, better recognize themselves as persons who are fulfilling their duty to the family and community, rather than as *carers*. Although the problem of identifying as a *carer* is not unique to South Asian communities, it can hinder access to vital support and resources [38] and may be particularly pertinent to these communities.

Although we were not able to recruit participants from a diverse range of ethnic and cultural groups, the insights that emerged from those that did participate illustrated the need for further, more detailed exploration of the role culture and ethnicity may play in the uptake of such technologies.

Although the sample size was relatively small, it is worth acknowledging that there was a high level of coherence and conformity among the data that were collected through a variety of techniques. Data saturation was reached in discussions about the usefulness of the platform.

### Comparison With Previous Studies

Although the use of digital technologies is increasing throughout all age groups across the population, there remains greater fear and anxiety among older adults toward using them, as well as a lack of confidence in their own skills and abilities to do so [39]. Our study suggests that older carers recognize that online technologies are potentially valuable and relevant. Our findings demonstrate that members of this this group are willing to learn how to navigate through a well-designed and tailored platform, such as Care Companion. This is in line with the model of technology acceptance proposed by Barnard et al [40].

Participants in our study indicated that an in-person introduction to Care Companion, such as through brief individual or group training sessions, could help increase the understanding and uptake among older carers. Studies have shown that supportive environments can have a powerful role for encouraging the use of digital technologies, whether through step-by-step guidance, offering a friendly space to use trial and error methods, or through providing an instruction manual [40]. Preferably, this should involve a user-centered model where an individual’s unique characteristics and needs are taken into consideration [41].

Concerns about online security and confidentiality are reported in other studies [42,43], where older adults report fear that their personal data may be misused and manipulated [42]. Our study shows that older carers are aware of these risks and are eager to understand how their personal information is being stored and used. They were concerned that if they uploaded personal information this would compromise their safety and there might be the possibility of other people reading and accessing their private notes. This highlights how important it is for platforms, such as Care Companion, to be unambiguous and transparent about how information is stored and that this is presented clearly in simple language. Although 2-factor authentication is in place to help protect users’ information, for Care Companion, this entailed users receiving a phone call with a 4-digit pin code whenever they logged in from a different internet protocol address; this verification process was seen as cumbersome and off-putting. There is a need for authentication mechanisms to be accessible and inclusively designed for a broad range of users [44]. As a result, Care Companion’s 2-factor authentication process has now been modified to include a number of changes to make it easier to hear and understand the automatic call back. The introduction of an *I’m ready* button to allow users time to find their telephone or a pen and paper on which they could write down the code, has also been added.

The journal feature of Care Companion was widely considered to be the most useful aspect of the platform and which would encourage the site’s continuous use. Writing expressively about emotionally triggering events is recognized as having positive effects on physical and mental well-being [45,46]. This may help the carers to understand, regulate, and process difficult emotions and so shape affective and cognitive state, as well as serve as an aide-memoire when explaining issues to a health or social care provider [46].

### Conclusions

Exploring the acceptability and aspects of use of Care Companion has been an informative and important step between the coproduction process and the wider realization and evaluation of the platform. A number of insightful lessons emerged, illustrating the importance of careful user acceptance testing [15].

The key findings identified during the coproduction phase of Care Companion’s development were reinforced by this study. These include the need for resources aimed specifically at carers (instead of care recipients); importance of personalized information; and the value of having a journal. This acceptance testing further highlighted issues that had not previously been identified during the coproduction phase, which include the importance of transparency for security and data usage; minimizing barriers to initial registration; and offering demonstrations to support a wider uptake by people with low technological literacy. In addition, this study underscores the need to develop a culturally sensitive approach to promoting Care Companion that works in partnership with and reflects the diversity of the local population.

The evidence from our study is relevant to the wider development of digital interventions for carers and is now informing the strategy for a full area-wide implementation of Care Companion.

## Appendix

Multimedia Appendix 1 Details of data collection.

Multimedia Appendix 2 Interview schedule–think-aloud interviews.

## Abbreviations

IT: information technology
UTAUT: Unified Theory of Acceptance and Use of Technology
