# Supplementary material for: Exploring the Perceived Usefulness and Ease of Use of a Personalized Web-Based Resource (Care Companion) to Support Informal Caring: Qualitative Descriptive Study
Source: JMIR Aging. 2019 Aug 20;2(2):e13875. doi: 10.2196/13875 (PMC6816311; doi:10.2196/13875)
Supplement: Multimedia Appendix 1 [file aging_v2i2e13875_app1.pdf]

Appendix 1

Details of data collection

Phase	Identifier	Data Collection Method	Recruitment strategy	Details	Gender	Number of Participants	Details about caring responsibilities
Phase 1	P1-1	Think aloud Interview	Rural Café for supporting older people and their carers	researcher guided "think-aloud" interviews while navigating prototype	female	1	lived separately from person needing care
	P1-2	Think aloud Interview	Rural Café for supporting older people and their carers	researcher guided "think-aloud" interviews while navigating prototype	female	1	Lived with person needing care
	P1-3	Think aloud Interview	Rural Café for supporting older people and their carers	researcher guided "think-aloud" interviews while navigating prototype	male	1	Lived with person needing care
	P1-4	Think aloud Interview	Rural Café for supporting older people and their carers	researcher guided "think-aloud" interviews while navigating prototype	female	1	Lived with person needing care
Phase 2	FG 1	Focus Group	Rural Café for supporting older people and their carers	One researcher spoke with three participants and introduced the prototype, and led a general discussion. Participants were invited to trial the platform for a couple of weeks and then participate in an interview.	3 female	3	All three participants were carers for somebody they lived with (parent or spouse)

	FG 2	Focus Group	Local Parkinson's disease charity	Two researchers attended a large group meeting (more than 30 attendees) and gave a general presentation about the Care Companion. Following the presentation participants asked questions and gave comments about the platform. Following the large group discussion, a smaller group of interested participants (6) came and engaged with the prototype on tablet devices in a session facilitated by both researchers. Both researchers took notes.	Large group - mixture of males and females	37	The group was a mixture of people in care and their carers
	FG 3	Focus Group	Local Parkinson's disease charity	Two researchers attended a smaller group meeting with interested participants who were not able to attend the first large group meeting. One researcher gave a short presentation and facilitated a group discussion about the platform. The second researcher made field notes while the first led the discussion. Following the discussion, participants were invited to use Care Companion for a couple of weeks and then participate in an interview. Those interested signed consent forms and provided the researchers with contact details.	3 male, 4 female	7	The group was predominantly made up of carers, one participant identified as caring for himself

	FG 4	Focus Group	South-Asian Carer Network	One researcher made contact with a local South-Asian Carer group and ran a focus group with three participants.	1 male, 2 female	3	All lived with person needing care
	P2-1	Interview	Referred by carer panel member	Telephone interview conducted after 2 weeks of using Care Companion.	male	1	Lived with and caring for spouse for 2 years at time of interview
	P2-2	Interview	Rural Café for supporting older people and their carers	Following a focus group telephone interview conducted after 2 weeks of using Care Companion.	female	1	Caring for 4 years providing daily care at the time of interview
	P2-3	Interview	Recruited through invitation sent to dementia support group	Following a focus group telephone interview conducted after 2 weeks of using Care Companion.	male	1	Living with and caring for spouse for 4 months at time of interview
	P2-4	Interview	Rural Café for supporting older people and their carers	Following a focus group telephone interview conducted after 2 weeks of using Care Companion.	female	1	Living with and caring for parent for 7 months at time of interview
	P2-5	Interview	Local Parkinson's disease charity	Following a focus group telephone interview conducted after 2 weeks of using Care Companion.	female	1	Living with and caring for spouse for 6 years at time of interview
	P2-6	Interview	Local Parkinson's disease charity	Following a focus group telephone interview conducted after 2 weeks of using Care Companion.	male	1	Living with a condition, cared for self and supports their carer to care for them
