# Supplementary material for: Exploring the Perceived Usefulness and Ease of Use of a Personalized Web-Based Resource (Care Companion) to Support Informal Caring: Qualitative Descriptive Study
Source: JMIR Aging. 2019 Aug 20;2(2):e13875. doi: 10.2196/13875 (PMC6816311; doi:10.2196/13875)
Supplement: Multimedia Appendix 2 [file aging_v2i2e13875_app2.pdf]

Appendix 2

Interview schedule – Think-Aloud Interviews

Introduction: Introduce the Care Companion

Instruction: Set up mock profile with the participant. Provide guidance if needed.

Questions:

1) What did you think about the process of setting up a profile?
2) How easy were the questions to answer?
3) Was there anything you would be uncomfortable including in your own profile?

Instruction: Encourage participant to navigate through the Care Companion website sections freely.

Questions to be asked whilst participant is navigating sections:

1) Why did you click on that part of the Care Companion? (e.g. FAQ, glossary, resource, journal, contacts, to dos, mood monitor)
2) What did you expect to find when the page opened?
3) Was what you found what you expected? If not why?

Instruction: If they need help either going back to where they were, or working out where to go next, ask them about this:

1) What was difficult about navigating to the next page/section?
2) What caused you to get stuck on this page?

End of interview:

1) Do you have any further questions or comments about the Care Companion?

Interview Schedule – Follow-up Telephone Interviews

Introduction: check participation understood:

1) Willing to participate and consent signed-audio-recorded
2) Understands can end interview at any time
3) Confidentiality explored- what will happen to the information

Background:

1) How long have you had a caring role?
2) What is your relationship to your cared for person?
3) Do you have any experience of using computers and the internet?

- Prompts: e.g. at home, at work, or at the library – explore access to computer, tablet or smartphone

4) Do you use the internet for information and advice? What kind of websites do you use?
5) Prompts: How much do they search for resources, do they find it easy/difficult to navigate the web

Care Companion:

1) What is your general view of the Care Companion platform?
2) Prompts: it's overall usefulness, easy/difficult to complete, did it meet your expectations?
3) Which parts of it did you use most? Which parts worked well?

   - Prompts: Easiest/ most useful parts? What did you like about the CC? If needed, ask for specific sections: the resource library, the journal, the mood monitor, the contacts, to dos and notifications, FAQs, glossary

4) Which parts of the CC did not work well for you?

   - Prompts: Which parts only used a only little/not at all? Not interested in? Any technical difficulties or navigational difficulties?

5) Is there anything that you would have liked to see and use in the Care Companion that was missing?

   - Prompts: Both in terms of actual resources/themes, and sections within the Care Companion

6) How would you use the Care Companion in your day-to-day life?

   - Prompts: How could it help you care better for your cared for, and look after yourself better?

7) Do you have any other comments to make?
